# Neurorestorative Therapy for Stroke

**DOI:** 10.3389/fnhum.2014.00382

**Published:** 2014-06-27

**Authors:** Jieli Chen, Poornima Venkat, Alex Zacharek, Michael Chopp

**Affiliations:** ^1^Department of Neurology, Henry Ford Hospital, Detroit, MI, USA; ^2^Department of Physics, Oakland University, Rochester, MI, USA

**Keywords:** BMSC, HUCBC, neurorestoration, microRNA, niaspan

## Abstract

Ischemic stroke is responsible for many deaths and long-term disability world wide. Development of effective therapy has been the target of intense research. Accumulating preclinical literature has shown that substantial functional improvement after stroke can be achieved using subacutely administered cell-based and pharmacological therapies. This review will discuss some of the latest findings on bone marrow-derived mesenchymal stem cells (BMSCs), human umbilical cord blood cells, and off-label use of some pharmacological agents, to promote recovery processes in the sub-acute and chronic phases following stroke. This review paper also focuses on molecular mechanisms underlying the cell-based and pharmacological restorative processes, which enhance angiogenesis, arteriogenesis, neurogenesis, and white matter remodeling following cerebral ischemia as well as an analysis of the interaction/coupling among these restorative events. In addition, the role of microRNAs mediating the intercellular communication between exogenously administered cells and parenchymal cells, and their effects on the regulation of angiogenesis and neuronal progenitor cell proliferation and differentiation, and brain plasticity after stroke are described.

## Introduction

Stroke is one of the leading causes of mortality, long-term disability, and morbidity. Current stroke treatments have mostly targeted early neuroprotection, utilizing therapeutic agents designed to prevent or reduce cell damage from ischemia. These agents have provided promising results in animal stroke models. However, except for thrombolysis with tissue plasminogen activator (tPA), to-date, all Phase III clinical trials utilizing them have failed to provide therapeutic benefit. For now, tPA is the only FDA approved pharmacological therapy for acute ischemic stroke. Based on the European Cooperative Acute Stroke Study (ECASS III) (Cronin, 2010; Carpenter et al., 2011), tPA was therefore approved for thrombolytic therapy of acute ischemic stroke for selected patients when therapy starts within 4.5 h of stroke onset. However, treatment with tPA is limited by this narrow time window, as well as by an increased risk for intracranial hemorrhage. Consequently, only 4–7% of patients in US suffering a stroke receive thrombolysis with tPA (Katzan et al., 2004; Schwammenthal et al., 2006; Weimar et al., 2006). After decades of research focused on acute neuroprotection and the failure of clinical trials to overcome this barrier, the National Institutes of Neurological Disease and Stroke (NINDS) Stroke Progress Review Group in 2006 and in 2011 identified delayed neurorestoration after stroke as a major priority for stroke research (Grotta et al., 2008; NINDS, 2012).

Cell-based and pharmacological restorative therapies are promising approaches for the treatment of stroke (Modo et al., 2002a,b; Savitz et al., 2003; Watson et al., 2003; Willing et al., 2003). Cell-based therapy, e.g., bone marrow stromal cells (BMSC) or human umbilical cord blood cells (HUCBCs), when administered intravenously after stroke improves neuroplasticity and neurological outcome through the up-regulation of restorative processes such as: neurogenesis, angiogenesis, and oligodendrogenesis in the post-ischemic brain (Chen et al., 2001a). Among pharmacological agents, which promote neurological recovery when administered subacutely, Niaspan, a slow-releasing vitamin B3 drug has shown promise as a neurorestorative agent (Chen et al., 2007). In addition, Niaspan extends the therapeutic window for tPA therapy (Chen et al., 2001a; Shehadah et al., 2011). Recently, microRNAs (miRNAs), each of which may regulate the translation of hundreds of genes and thereby act as molecular master switches, have been shown to mediate BMSC cell therapy for stroke (Juranek et al., 2013) and control neuronal progenitor cell proliferation and differentiation, and will therefore be discussed here (Lim et al., 2010; Wang et al., 2013). This review paper will also focus on molecular mechanisms promoting neurorestorative effects (angiogenesis, neurogenesis, and oligodendrogenesis) after cerebral ischemia, which underlie the restorative effects of cellular and experimental pharmacological approaches for the treatment of stroke.

## Biological Bases for Neurorestorative Therapy Post-Ischemia

Neurorestoration post-stroke is achieved by enhancing neurogenesis, angiogenesis, and oligodendrogenesis, which in concert promote neurological recovery (Chen et al., 2003b, 2006, 2010; Chopp et al., 2009; Hermann and Chopp, 2012). Neurogenesis, the generation of new parenchymal cells from neural stem and progenitor cells, stimulates plasticity, oligodendrogenesis, restores neuronal signal transduction, and promotes myelination (Skihar et al., 2009), Vascular remodeling (angiogenesis and arteriogenesis) increases cerebral blood flow (CBF) perfusion and mediates the generation of important restorative trophic factors and proteases and thereby helps to establish a hospitable environment for neurite outgrowth, remyelination, and in general for the resident cells (Chen et al., 2009).

### Vascular remodeling (angiogenesis and arteriogenesis)

Recent findings concerning the pathophysiological events following acute ischemic stroke suggest that angiogenesis plays a critical role in improving long-term recovery of patients (Arenillas et al., 2007; Navarro-Sobrino et al., 2011). Typically, elderly patients tend to have lower levels of new vessel formation following stroke, which may be associated with lower rates of functional recovery (Allen, 1984; Granger et al., 1992). Maintenance of neural function is critically dependent upon regulation of CBF (Pratt et al., 2004). Angiogenesis and arteriogenesis reestablish functional microvasculature in the ischemic border zone (IBZ), creating a microenvironment hospitable for neuronal plasticity, which can lead to functional recovery (Plate, 1999; Chen et al., 2003b; Renner et al., 2003). After stroke, patients who have a higher cerebral blood vessel density do better and survive longer than patients having lower vascular density (Krupinski et al., 1994; Wei et al., 2001). Clinical improvement shortly after stroke also correlates with the presence of arteriolar collaterals (arteriogenesis). Stroke mortality increases in the absence of significant collateralization (Christoforidis et al., 2005). It is therefore reasonable that stimulating angiogenesis and arteriogenesis may be an effective treatment strategy for stroke patients.

Angiogenesis leads to mature and functional blood vessels, which is a therapeutic goal. Angiogenesis refers to the biological process resulting in the growth of new blood vessels, branching off from pre-existing vessels. Hypoxia and tissue ischemia are the main physiological stimuli for angiogenesis (Dor and Keshet, 1997). Endothelial cell proliferation, migration, and sprouting of new vessels from existing vessels toward the site of ischemic brain injury are stimulated when angiogenic factors bind to specific receptors located on brain endothelial cells (Greenberg, 1998). This sprouting of endothelial cells leads to the formation of tube-like vascular structures. The initial vascular plexus during angiogenesis forms mature vessels via branching, pruning, sprouting, as well as the promotion of differential growth of endothelial cells, and the recruitment of supporting cells, such as pericytes and smooth muscle cells (SMCs) (Folkman and D’Amore, 1996; Risau, 1997). Angiogenesis and vascular maturation are regulated by many factors, such as, Angiopoietin-1 (Ang1)/Tie2 system (Patan, 2004), basic fibroblast growth factor (bFGF), endothelial nitric oxide synthase (eNOS), platelet-derived growth factor (PDGF), and vascular endothelial growth factor (VEGF) (Greenberg, 1998; Lutsenko et al., 2003; Matsui and Tabata, 2012). To determine the optimal window for the initiation of angiogenic therapies, a rigorous timetable of angiogenic event steps must be drawn. Angiogenesis takes place in the penumbra of human ischemic brain hours after initial onset and continues to be present weeks after ischemic onset (Krupinski et al., 1994). Nitric oxide (NO) initiates vasodilation and is considered the first step in angiogenesis (Carmeliet, 2000). Combining the vasodilation effect of NO with the increase in VEGF expression, which increases vascular permeability allows extravasation of plasma proteins that lay down a provisional scaffold for the migration of endothelial cells for vascular sprouting. The second step involves the dissociation of SMCs and loosening the extracellular matrix, which enwraps the mature vessel. Angiopoietin-2 (Ang2), an inhibitor of Tie2 signaling, may be involved in facilitating the detachment of pericytes from endothelial cells, while the matrix metalloproteinase (MMP) family of proteinases degrade matrix molecules and further weakens vascular integrity (Feng et al., 2009). Once the path of sprouting has been established, proliferating endothelial cells migrate to distant sites. During this time, an array of molecular signals, including VEGF, VEGF receptors, and placental growth factor, work in synchrony to guide this process. Once new blood vessel networks are formed, Ang1, which activates Tie2 receptors, helps to stabilize networks initiated by VEGF. The angiogenic process is tightly regulated by growth factors and the up-regulation of specific growth factors that dictate event progression (Greenberg, 1998; Lutsenko et al., 2003).

Arteriogenesis adapts existing systems of vessels into functional ancillary conduits for blood flow to tissues distal to the site of occlusion of large, peripheral conduit arteries (Buschmann and Schaper, 1999; Schaper and Buschmann, 1999). This process shares characteristics with angiogenesis, though different pathways lead to arteriogenesis. The main differences between arteriogenesis and angiogenesis reside in the development of collaterals from existing arterioles, which are activated by the large pressure differences between perfusion territories, which produce high intravascular shear stress (Erdo and Buschmann, 2007). The attraction, adhesion, and activation of circulating cells such as monocytes, T-cells, and basophils, are responsible for a large percentage of the vascular–arteriolar growth and remodeling (Schaper and Buschmann, 1999). The majority of growth factors and proteolytic enzymes are secreted by monocytes, and they allow SMCs to migrate and divide (Scholz et al., 2001). Arteriogenesis results in the formation of new arterioles, which are believed to occur when SMCs coat pre-existing capillaries, which are then transformed into larger diameter channels (Buschmann and Schaper, 2000; van Royen et al., 2001). Arteriogenesis is a decisive process of altering existing vessels into functional collateral conduits to bypass occlusions of large peripheral conduit arteries and restore the supply of oxygen-enriched blood to tissues (Seetharam et al., 2006).

Angiogenesis and arteriogenesis foster functional restoration after neurological injury by inducing brain plasticity (Risau, 1997; Plate, 1999; Cramer and Chopp, 2000; Cairns and Finklestein, 2003; Renner et al., 2003; Landers, 2004). Vascular remodeling promotes neurorestoration, which is the goal of all neurorestorative therapies (Hurtado et al., 2006). Generating new blood vessels post-ischemia promotes neurorestorative processes such as neurogenesis and synaptogenesis, which foster improved functional recovery.

### White matter and axonal remodeling and oligodendrogenesis

In humans, after stroke or any acute injury to the central nervous system (CNS), functional recovery is highly limited, leaving many survivors with life-long neurological deficits. The inability to completely restore function in these individuals can be partially attributed to inadequate axonal regeneration and neuroplasticity (Walmsley and Mir, 2007). Preclinical studies demonstrate that remodeling of the axons starts 2–3 weeks post-stroke (Liu et al., 2009, 2010). Successful axonal outgrowth in the adult CNS is critical to brain repair processes after stroke (Hou et al., 2008). Surviving cortical neurons in the peri-infarct motor cortex experience axonal sprouting, which can restore connections in the brain (Carmichael et al., 2001; Carmichael, 2003; Dancause et al., 2005). Thus, axonal remodeling in the corticospinal system may contribute to spontaneous functional recovery following stroke (Liu et al., 2009).

Oligodendrogenesis and remyelination play a crucial role in behavior and functional restoration post-ischemia. Oligodendrocytes (OLs) produce the myelin sheaths, which wrap around axons, facilitating nerve conduction. Oligodendrocyte progenitor cells (OPCs) differentiate into mature OLs. OLs are highly vulnerable to ischemic stress, because white matter has lower blood flow than gray matter, and deep white matter has little collateral blood supply (Back et al., 2002). OL damage leads to demyelination, which contributes to neurological and behavior function deficits. Mounting evidence suggests that inflammatory response post-ischemia is especially detrimental to white matter cohesion through the up-regulation of matrix MMPs (Chen et al., 2011). MMP-9 and MMP-2 have both been shown to increase white matter lesions (Chen et al., 2011). In the rodent transient middle cerebral artery occlusion model (MCAo), the number of OLs decreased between 24 and 48 h after ischemia and increased 1–2 weeks after reperfusion in the peri-infarct cortex (Tanaka et al., 2001). Ischemic damage of OL results in demyelination, contributing to neurological and behavioral functional deficits. Although mature OLs are considered post-mitotic and unable to proliferate, the white matter contains an abundance of OPCs that respond to ischemic injuries (Gensert and Goldman, 1997; McTigue and Tripathi, 2008). Evidence of oligodendrogenesis post-ischemic stroke has been demonstrated (Iwai et al., 2010).

### Neurogenesis and synaptogenesis

Neurogenesis and synaptogenesis contribute to post-stroke functional improvement. Adult mammalian neurogenesis takes place in the subgranular zone (SGZ) and subventricular zone (SVZ) of the hippocampus (Shehadah et al., 2010a). Under normal conditions, the neural progenitor cell (NPC) population is maintained through tightly regulated cell apoptosis and proliferation (Shehadah et al., 2010b). After ischemic stroke onset in rats, the population of NPCs significantly expands in the SVZ, and neuroblasts are systematically recruited and differentiate into mature neurons, astrocytes, and OLs in the migratory target of the ischemic penumbra region (Parent et al., 2002). Forty-eight hours post-ischemic stroke, NPC proliferation is up-regulated in the SVZ in the adult rodents by shortening the cell-cycle length from 19 h in non-stroke SVZ cells to 15.3 h in stroke SVZ cells (Zhang et al., 2006). The decrease in the duration of the cell cycle is mainly due to a reduction in the G1 phase (Zhang et al., 2006). A larger percentage of progenitor cells from the stroke SVZ re-enter the cell cycle after mitosis than cells from the non-stroke SVZ (Zhang et al., 2006). Fourteen days post-ischemia, cell-cycle lengthening results in daughter cells, which exit the cell cycle and differentiate into neural cells (Zhang et al., 2008). Above evidence suggests that neurorestoration follows a tightly regulated sequence of events with the initial step of NPC proliferation followed by differentiation of these progenitor cells into mature neural cells. Following an ischemic insult, neurogenesis in the SVZ is enhanced and precursors migrate to the IBZ and differentiate into region-specific neural phenotypes (Parent et al., 2002). Cell therapies enhance this endogenous neurogenesis, migration, and differentiation of neural cells and promote neurological recovery (Chen et al., 2003a, 2013; Munoz et al., 2005; Bao et al., 2011; Zhang et al., 2012b; Gutierrez-Fernandez et al., 2013).

Synaptogenesis, the process of formation of new synapses, can be enhanced by angiogenesis, as there is enhanced oxygen supply to tissue via blood vessels (Zhang and Chopp, 2009). Increased expression of Synaptophysin, a pre-synaptic vesicle protein and an indicator of synaptogenesis (Stroemer et al., 1995; Ujike et al., 2002), has been observed in cell therapy treatments (Zhang et al., 2012b; Gutierrez-Fernandez et al., 2013) with a correlated improvement in post-stroke functional outcome. From our work in neurorestorative therapies for stroke, we have found that all agents and cell types that are effective in promoting recovery of neurological function evoke common responses in cerebral tissue (Chen et al., 2001a; Li et al., 2001a, 2002; Zhang et al., 2001, 2002; Wang et al., 2004).

### Neurovascular niche

The neurovascular unit is a conceptual model that describes functional interactions and signaling between neurons, capillaries, and glia in the brain. Since stroke inflicts both neural and vascular damage, therapeutic interventions targeting the vascular neural network warrant investigation as a fundamental component for post-stroke neurorestoration (Zhang et al., 2012a). Angiogenesis and neurogenesis in the neurovascular niches of the CNS play key roles in recovery. Based on *in vivo* and *in vitro* murine models of sublethal hypoxia, it has been suggested that the neurovascular niches of the CNS, in response to hypoxia, trigger HIF-1α-mediated responses (Madri, 2009). HIF-1α is modulated in part by NO, modulates brain-derived neurotrophic factor (BDNF), VEGF, and stromal cell-derived factor 1 (SDF-1), and induces their autocrine and paracrine signaling, which in turn mediates endothelial cell and neural stem cell survival and proliferation (Madri, 2009). Thus, the optimization of the expression levels of hypoxia-induced induction of HIF-1α and its downstream signaling components BDNF, C–X–C chemokine receptor type 4 (CXCR4), Neuropilin-1 (Nrp-1), NO, SDF-1, and VEGF may maximize recovery (Madri, 2009). In a model of focal cortical stroke, migration of newly formed neurons from the SVZ to cortex, neurogenesis from a glial fibrillary acidic protein (GFAP)-expressing progenitor cells in the SVZ, and migration of neuroblasts to a neurovascular niche in peri-infarct cortex can improve behavioral recovery post-stroke (Ohab et al., 2006). Behavioral recovery is thus, attributed to a process linking neurogenesis and angiogenesis by growth factors and chemokines and to the trophic action of SDF-1 and Ang1, which are up-regulated by blood vessels within the neurovascular niche (Ohab et al., 2006).

## Neurorestorative Treatment of Stroke with Cell-Based Therapy

### Human umbilical cord blood cells

Human umbilical cord blood cells hold great promise as therapeutic agents, since they are easy to isolate without serious ethical and technical problems. HUCBCs are a rich source of mesenchymal and hematopoietic progenitor cells (HPCs). The number of highly proliferative HPCs in bone marrow is equaled or exceeded by those found in HUCBC (Almici et al., 1995). HUCBCs induce strong immunomodulatory properties by the host and yet remain weakly immunogenic themselves (Vendrame et al., 2006; Nikolic et al., 2008). As observed in an animal stroke model, HUCBCs inhibit the pro-inflammatory T helper cell type 1 (Th1) response, while promoting a strong anti-inflammatory T helper 2 (Th2) response (Vendrame et al., 2004; Nikolic et al., 2008). Numerous studies having shown that HUCBC treatment of rodents does not elicit GVHD (Graft Versus Host Disease), a leading cause of death in patients that have received stem cell transplants (Li et al., 2001b; Lu et al., 2002; Henning et al., 2004; Hu et al., 2006). Patients who receive HUCBC transplants from a relative are significantly at a lower risk of GVHD, and are less likely to reject the transplant compared to either bone marrow or peripheral blood stem cells (Takahashi et al., 2007; Morgado et al., 2008). Factors that may be beneficial to the host brain *in vivo* are secreted by HUCB-derived mononuclear cells as they proliferate and differentiate (Neuhoff et al., 2007). Umbilical cord blood can provide a significant number of stem/progenitor cells, for hematopoietic as well as other tissue-specific lineages, including nervous tissue (Li et al., 2001b; Kozlowska et al., 2007). HUCBCs, when intravenously (i.v.) administered, migrate selectively to the ischemic area in the brain, enhancing functional recovery post-stroke (Chen et al., 2001b; Li et al., 2001b; Zhang et al., 2011).

The mechanism of transplanted HUCBC-induced functional benefit after stroke is not clear. The beneficial effects of HUCBC treatment may be due to multiple causes, such as improved cell survival, increased angiogenesis, nerve fiber reorganization, reduced inflammation, and trophic actions, among other restorative events (Vendrame et al., 2006; Arien-Zakay et al., 2011; Liu et al., 2014).

#### Anti-inflammatory effects

Beneficial effects include reduction in the extent of ischemic damage, and CD8+ T-cell counts in MCAo rat model (Vendrame et al., 2006). HUCBC treatment at 48 h post-stroke significantly decreased infiltration of granulocytes and monocytes and reduced astrocytic and microglial activation in the parenchyma (Newcomb et al., 2006). Functional recovery from permanent MCAo was also seen upon intravenous HUCBC administration in spontaneously hypertensive rats (Miller et al., 2013). While both human CD34^−^ and CD34^+^ cells derived from HUCB were found to be equally competent in stroke treatment, easy attainability of CD34^−^ cells in comparison to purified CD34^+^ cells, makes it a promising source for cell-based therapies for humans (Miller et al., 2013). HUCBC administration suppresses pro-inflammatory factor expression, including cytokines, CD45/CD11b-, CD45/B220-positive (+) cells, nuclear factor-κB (NF-κB) DNA binding activity (Vendrame et al., 2005), tumor necrosis factor-α (TNF-α) (Chen et al., 2008), and suppression of pro-inflammatory isolectin binding cells (Leonardo et al., 2010), which may lead to functional and anatomical recovery by attenuating neuroinflammation and inducing neuroprotection (Vendrame et al., 2005; Leonardo et al., 2010).

#### Trophic factor effects

The therapeutic benefits of HUCBC treatment likely derive from enhancement of endogenous brain recovery mechanisms (Chen et al., 2001b). Only a small percentage of HUCBCs employed to treat stroke expresses proteins phenotypic of neural-like cells and functional recovery occurs within days after HUCBC administration (Chen et al., 2001b). Additionally, the number of HUCBCs administered intravenously that enter the brain was small, and the tissue that was replaced would make up no more than a cubic millimeter. HUCBC treatment of stroke also elevated levels of glial cell-derived neurotrophic factor (GDNF), nerve growth factor (NGF), and BDNF, thus, trophic factor-mediated mechanisms contribute to improved behavioral outcome, rather than cell replacement (Yasuhara et al., 2010). HUCBCs contain many hematopoietic colony-forming cells (CFS) (Nakahata and Ogawa, 1982), as well as produce IL-11 and thrombopoietin (Suen et al., 1994). CSF-1, a hematopoietic cytokine, is a CNS growth factor (Berezovskaya et al., 1995). As such, it is likely that HUCBCs act as sources of trophic factors (Chen et al., 2008). In spinal cord injury (SCI), treatment with HUCBCs in rats increased serum levels of GDNF, IL-10, and VEGF, which may contribute to the observed beneficial effects (Chen et al., 2008). Cytokines and growth factors in murine NPCs are survival and/or differentiation factors (Mehler et al., 1993) that may play a critical role in neural tissue proliferation or differentiation (Cairns and Finklestein, 2003). When HUCBCs are administered intravenously and migrate to the injured tissue, they may function as a site of trophic factor production, which bypasses the blood–brain barrier (BBB). Therefore, functional benefit of i.v. administration of HUCBC, likely, does not derive from cellular replacement of tissue but from trophic factor expression by the injected cells and/or induced by the injected cells within the parenchymal tissue (Chen et al., 2008; Liu et al., 2014).

Studies also point at OL protection and survival as a means of HUCBC-induced neuroprotection (Rowe et al., 2010, 2012). Umbilical cord blood cell-derived CD34+ cells promote angiogenesis, neurogenesis (Chen et al., 2013), neuronal regeneration resulting in enhanced neovascularization (Taguchi et al., 2004), which amplifies the neurorestorative effects and improves functional recovery after stroke. But without reducing infarct volume and, necessarily, providing neuroprotection (Nystedt et al., 2006).

### Multipotent mesenchymal stem cells

Mesenchymal stem cells (MSCs) can be isolated from bone marrow (most common), cord blood cells, placenta, muscle, skin, and even liposuction fat. In this review, we will focus on MSCs derived from bone marrow. BMSCs are constituted by a heterogeneous collection of mesenchymal stem and progenitor cells. Human BMSCs (hBMSCs) can transdifferentiate into neural and mesodermal cell lines (Friedenstein et al., 1968, 1987; Bianco and Gehron Robey, 2000). In animal models, post-stroke hBMSCs transplantation improves sensory–motor function (Chen et al., 2001a; Zhao et al., 2002; Chen and Chopp, 2006; Weng et al., 2008; Huang et al., 2013b), enhances synaptogenesis (Weng et al., 2008), stimulates nerve regeneration (Tohill et al., 2004), decreases tPA-induced brain damage (Liu et al., 2012), and can mediate immunomodulatory effects and reduce inflammation (Yoo et al., 2009).

BMSC transplantation induces neurorestorative effects and ameliorates neurological functional deficits after stroke (Chen et al., 2001a, 2003a; Shen et al., 2007). We have demonstrated that BMSC therapy can reduce neurological functional deficits when administered intravenously at 1 or 7 days after stroke (Chen et al., 2001a) and even at 1 month after stroke (Shen et al., 2007). Transplanted adult BMSCs migrate to damaged tissue in brain and decrease post-stroke functional deficits (Chen et al., 2001a). Migration may be aided by the disruption of BBB enabling selective entry of BMSCs into ischemic brain compared to normal cerebral tissue. Neurological benefit is derived mainly by triggering the release of growth and trophic factors, as a very small percentage of cells migrate, differentiate, and contribute toward neuroprotection/restoration. BMSCs trigger the release of growth factors in ischemic tissue that can initiate cell repair mechanisms, enhance cell proliferation in SVZ, and reduce apoptosis and neuronal death/damage in ischemic region (Li et al., 2002).

The mechanisms of action of BMSC treatment of stroke significantly differ from those initially targeted for progenitor and stem cells. Progenitor and stem cells are placed in the injured brain, they are designed to replace injured and dead tissue (Riess et al., 2002). It is likely that BMSCs also contain a stem-like subpopulation of cells that can differentiate into brain cells (Zhang et al., 2009; Shichinohe et al., 2010; Ding et al., 2011). However, this is a minor subpopulation of BMSCs where only a minute percentage assumes a parenchymal cell phenotype, and do not contribute to functional recovery (Li et al., 2002).

Transplanted BMSCs, by releasing soluble trophic factors and cytokines, promote endogenous repair of neurologically damaged tissues (Hardy et al., 2008). We and others have shown that exogenous cells produce various factors, and more importantly, stimulate neuroprotective (Chen et al., 2003a) and neurorestorative factor production in parenchymal cells. Intravenously administered BMSCs used to treat stroke or CNS disease produce trophic factors and stimulate parenchymal cells to express trophic factors (Chen et al., 2003b). BMSCs express mRNAs covering a wide range of angiogenic/arteriogenic cytokines that include Ang1, basic fibroblast growth factor-2 (FGF2), insulin-like growth factor (IGF), placental growth factor, and VEGF (Chen et al., 2003b; Zacharek et al., 2007). hBMSCs that had been cultured with extract from ischemic rat brain showed increased levels of BDNF, hepatocyte growth factor (HGF), and VEGF (Chen et al., 2002a,b). Intravenous administration of BMSCs leads to a time dependent release of neurotrophins and angiogenic growth factors like BDNF, VEGF, bFGF, NGF, HGF, and GDNF (Chen et al., 2002a,b, 2003b; Zacharek et al., 2007; Wakabayashi et al., 2010). These cytokines and growth factors have both autocrine and paracrine activities (Matsuda-Hashii et al., 2004), which are the molecular signals of the body uses to regulate differentiation, proliferation, and cell survival. GDNF promotes neurogenesis, endogenous cell repair mechanisms, neuroblast proliferation, and migration from the SVZ and decreases apoptosis (Kobayashi et al., 2006; Yuan et al., 2013). Intravenously injected BMSCs enter the brain and stimulate the local parenchymal cells, mostly astrocytes and endothelial cells, to produce growth factors promoting angiogenesis and vascular stabilization, which are partially mediated by Ang1/Tie2 and VEGF/Flk1 (Zacharek et al., 2007). Elevated IGF-1 mRNA expression was seen in cells subjected to ischemic stress and enhanced IGF-1 mRNA, IGF-1, and IGF-1R immuno reactive cells seen upon treatment with BMSCs, indicating that IGF-1-mediated self repair mechanism may contribute to the gain in neurological function (Wakabayashi et al., 2010).

BMSCs increase angiogenesis and arteriogenesis (Kinnaird et al., 2004; Zhu et al., 2011). BMSC conditioned cell culture media promotes integration and proliferation of endothelial cells and SMCs *in vitro*, and when injected directly into the hind limb of an ischemic mouse, this media enhanced collateral flow recovery and remodeling (Zhu et al., 2011). Treatment with autologous BMSCs significantly increased arteriogenesis and improved blood flow in the chronic limb ischemia model (Zhu et al., 2011). BMSCs selectively migrate to the site of injury, participate in angiogenesis and arteriogenesis, as well as induce a neovascular response that results in a significant increase in blood flow to the ischemic area, aiding the repair of the injured brain (Cui et al., 2009). Trophic and growth factor production is stimulated from angiogenic and arteriogenic vessels by BMSCs, which enhance brain plasticity and recovery of neurological function after stroke (Kinnaird et al., 2004). Therefore, it is possible to think of BMSCs as behaving like small biochemical “factories,” busily producing as well as inducing many cytokines and trophic factors in vascular and parenchymal cells that contribute to the improvement of functional outcome after stroke (Liu et al., 2014). Intracarotid BMSC transplantation also promotes white matter remodeling in the cortical IBZ and corpus callosum by increasing axonal sprouting and remyelination (Shen et al., 2006). Other beneficial effects observed upon BMSC treatment include enhanced structural neuroplasticity and increased axonal outgrowth from healthy brain tissue (Andrews et al., 2008).

#### miRNA on MSC-induced neurorestorative effects

microRNAs are short sequences of non-coding RNA (ca. 22 nucleotides) found in animals and plants, which regulate gene expression both transcriptionally and post-transcriptionally. miRNAs can regulate many genes, pathways, and biological networks, either acting alone or with other miRNAs. It appears that miRNAs act like molecular rheostats, fine-tuning many biological processes regulating tissue repair like angiogenesis, inflammation, hypoxia–response, and stem cell biology by affecting gene regulation (Sen, 2011). Therapies aiding in tissue repair based on miRNAs are beginning to hold promise as we learn how to control and manipulate cell-specific miRNAs (Juranek et al., 2013). However, using miRNAs as therapeutic targets still hold many challenges, due to possible delivery and potential off-target effects. Cell-based therapy may be an effective means to manipulate miRNA expression (Juranek et al., 2013). Cells delivered by intravenous injection release microvesicles that contain enriched miRNA that can in turn stimulate endogenous brain cells to express and release miRNA, ultimately promoting neurorestorative effects after stroke (Juranek et al., 2013).

BMSCs are abundantly present in our body and they secrete extracellular vesicles, which depending on parent phenotype, may carry miRNA, mRNA, proteins, lipids, etc (Katsuda et al., 2013) and transport miRNAs from cells of origin to target cells (Collino et al., 2011). miRNAs play a key role in BMSC neuronal differentiation during which miR-34a is downregulated. miR-34a mediates neuronal precursor motility, that is crucial for homing of stem cells to target tissue (Chang et al., 2011). In addition, miR-96, miR-124, and miR-199a regulate gene expression critical for differentiation of BMSCs, and were expressed differentially during adipogenic, chondrogenic, and osteogenic induction of hBMSCs (Laine et al., 2012). miRNAs regulate neurogenesis, mediate the trans-differentiation of MSCs into functional neurons and are also useful in restoration of lost or damaged neurons in neurological disorders (Lim et al., 2010). miRNAs, with their varied biological functions and regulatory capabilities, open new avenues and strategies for BMSC therapy and manipulation with potential therapeutic benefits.

Microvesicles provide bidirectional miRNA exchange between injured cells and BMSCs, which in turn facilitates neuronal differentiation and activation of regenerative pathways in injured cells. These extracellular vesicles can mediate intracellular communication and be manipulated to induce therapeutically beneficial effects. While in resting or active states, BMSCs secrete microvesicles, both microparticles and exosomes that can be manipulated to deliver miRNAs to enhance recovery of injured tissues (Wang et al., 2013). A novel treatment strategy for malignant glioma using miRNAs that are known to have anti-tumor properties (e.g., miR-146b), employs exosomes secreted by BMSCs transfected with miR-146b as a delivery vehicle to decrease tumor volume (Katakowski et al., 2013). Exposure to ipsilateral ischemic tissue extracts obtained from MCAo rats elevates miR-133b expression in BMSCs and in exosomes derived from BMSCs (Juranek et al., 2013). miR-133b transfer from BMSCs to neurons and astrocytes results in an increased miR-133b level, and when exposed to post-MCAo brain extracts for 72h, a significant increase in neurite branch number and total neurite length is observed. Neurite outgrowth also can be promoted by delivery of miR-133b to neurons and astrocytes by transfer from BMSCs via exosomes (Juranek et al., 2013). Manipulation the expression of miR-133b in BMSCs and thus, in their exosomes, regulates neurological recovery after stroke (Xin et al., 2013). These data clearly indicate that BMSCs mediate their functional benefit post-stroke, by the transfer of exosomes with active miRNAs to parenchymal cells. The miRNAs, and as shown specifically for the transfer of miR-133b, regulate their downstream targets, and thereby impact brain plasticity, and neurovascular remodeling to promote neurological recovery (Xin et al., 2013).

Therapeutic modulation of individual miRNAs generated by BMSCs, and either mimicking or antagonizing miRNA actions, may enhance BMSC therapeutic efficacy. For example, miR-126 is expressed in endothelial cells in blood vessels and capillaries, and mediate angiogenesis (Nikolic et al., 2010). Transplantation of BMSCs over-expressing miR-126, increases the release of angiogenic factors, improves resistance against hypoxia, and activates Notch ligand Delta-like-4, thereby enhancing functional angiogenesis in the ischemic myocardium and improves cardiac function (Huang et al., 2013a). Increased angiogenesis and improved cardiac function may be attributed to stimulation of AKT/ERK-related pathway (Chen and Zhou, 2011). Abnormal down-regulation of miR 146a has been implicated in mediating chronic inflammatory responses that interfere with wound healing in diabetic subjects (Xu et al., 2012). BMSC therapy for post-myocardial infarction, increases miR-146a expression and decreases the expression of pro-inflammatory factors. MiRNAs are also involved in nearly every aspect of the presumed repair mechanisms of BMSC-based therapies in myocardial infarction, such as neovascularization and stem cell differentiation (Wen et al., 2012). Hence, the use of miRNAs as novel regulators and therapeutic modulation of individual cardiovascular miRNAs of BMSCs have been proposed to improve therapeutic efficiency (Wen et al., 2012). Treatment of stroke with exosomes derived from BMSCs, i.e., without the parent BMSC, improves functional outcome, as well as enhances angiogenesis, neurogenesis, and neurite remodeling. This approach, of using purely exosomes derived from BMSCs, represents a potentially novel stroke treatment, with possible broad therapeutic applications (Xin et al., 2013).

### Stroke clinical trial for cell-based therapy

Isolating MSCs from bone marrow for transplantation is considered safe, having been widely tested in numerous clinical trials with encouraging results (Bang et al., 2005; Sykova et al., 2006). BMSC therapies are being evaluated via 79 registered clinical trial sites located throughout the world (Malgieri et al., 2010). Clinical trials conducted to study intravenous infusion of autologous BMSCs had promising results, showing that BMSCs appear to be a safe and are a feasible therapy for improving functional outcome in stroke patients (Bang et al., 2005; Suarez-Monteagudo et al., 2009; Lee et al., 2010). Autologous BMSCs were intravenously infused in a series of patients from South Korea suffering from cerebral infarcts in the middle cerebral artery (Bang et al., 2005). Studies, serial evaluations, and comparisons to control group (did not receive MSCs) for 1 year revealed that the treatment is safe and may improve functional recovery. A follow up long-term evaluation report recorded higher survival among treated patients than control group and revealed no significant side effects, indicating that autologous BMSCs delivered i.v. is safe and may improve functional recovery (Lee et al., 2010). A Phase I/II clinical trial has been initiated by researchers at University of California San Diego with other collaborators in which allogenic BMSCs are being evaluated to treat ischemic stroke (NCT01297413). A Phase I/II clinical trial in Spain revealed feasibility, safety, and improved neurological outcome in stroke patients transfused intra-arterially at 5 and 9 days after stroke with autologous bone marrow mononuclear cells (Moniche et al., 2012). During the follow up period of 6 months, no adverse effects, deaths, tumor formation, or stroke recurrence were reported, except for two isolated partial seizures at 3 months post treatment. From a study of a small group of ischemic stroke patients with infarcts in the middle cerebral artery region, it was found that the delivery of umbilical cord MSCs via intra-arterial catheterization is safe and may contribute to functional improvements (Jiang et al., 2012). There are several other ongoing studies to test the safety and efficiency of umbilical cord blood therapy, umbilical cord blood mononuclear cells, to treat stroke subjects (NCT01884155, NCT01673932).

## Niaspan Treatment Promotes Brain Plasticity after Stroke

There is a growing body of evidence that strengthens the link between brain high-density lipoprotein (HDL)-C metabolism and factors involved in synaptic plasticity. Scavenger receptor class B1 (SR-B1) binds HDL and facilitates α-tocopherol and cholesteryl esters transfer into cells from circulating HDL. Mice with knocked out scavenger receptor (SR-B1) exhibited deficient synaptic plasticity, as measured by long-term potentiation of the CA1 hippocampus region, which leads to impaired recognition and spatial memory (Chang et al., 2009). Furthermore, mice that lacked ATP-binding cassette transporter A1 (ABCA1) in the CNS exhibit reduced plasma HDL-C levels and altered synaptic morphology, including reduced synapse and synaptic vesicle numbers (Karasinska et al., 2009). Niacin is one of the most potent HDL-C promoter drugs used in the clinic. Niaspan, an extended release formulation of Niacin, may be effective in reducing neurological deficits post-stroke by promoting axonal remodeling, angiogenesis, and arteriogenesis (Chen et al., 2007, 2009; Cui et al., 2010; Yan et al., 2012).

Successful axonal sprouting and remodeling in the penumbra region is a critical step in nerve regeneration and brain repair. Niaspan, when administered 24 h after MCAo significantly up-regulates neuronal synaptic rewiring in the per-infarct region and restores connections between different cerebral areas after stroke (Cui et al., 2010; Yan et al., 2012). The increase in axonal density and synapse formation translates into long-term functional recovery after experimental stroke (Cui et al., 2010).

Niacin-induced increase in synaptic plasticity and axon growth may be mediated by the up-regulation in the BDNF–TrkB axis (Cui et al., 2010). In the mature nervous system, BDNF/TrkB plays an important role in regulating neuronal migration, differentiation, synaptic remodeling, and survival. Niacin treatment after stroke significantly increases BDNF/TrkB expression both in the ischemic brain and in primary cortical neuron (PCN) cultures. Furthermore, a TrkB inhibitor significantly decreases HDL and niacin-induced neurite outgrowth, which indicates that the BDNF/TrkB axis may mediate, niacin/HDL-induced synaptic plasticity and axon growth (Cui et al., 2010). In addition, the Ang1 molecular pathway also plays a partial role in Niaspan-induced axonal outgrowth (Yan et al., 2012). Niaspan significantly increases Ang1 expression. Ang1, in addition to being a promoter of angiogenesis and vascular maturation, is also a neurotrophic factor and promotes axonal outgrowth (Yan et al., 2012). In type 1 diabetes (T1DM) rats subjected to MCAo, Niaspan treatment attenuated Ang2 and increased Ang1 expression (Yan et al., 2012).

Early stroke recovery is linked to arteriogenesis (Christoforidis et al., 2005; Liebeskind, 2005). Occlusion of intracerebral arteries elevates fluid shear stress and thus primes the brain for arteriogenesis. Cellular interaction of the endothelial cells in the vascular wall with cytokines like monocyte chemoattractant proteins-1 (MCP-1), TNF-α, and cell adhesion molecules facilitates arteriogenesis, typically triggered by the development of elevated shear stress in the vessel (Hoefer et al., 2002). TNF-α is a pivotal modulator of arteriogenesis and the TNF-α-converting enzyme (TACE) is the primary protease responsible for pro-TNF-α activation (Chen et al., 2009). Treating stroke with Niaspan significantly increases CBF in the ischemic brain, as measured by magnetic resonance imaging (MRI), resulting in increased arterial diameter and proliferation of vascular SMC (VSMC). Treatment with Niaspan increases cultural arterial sprouting and VSMC migration *in vitro*. The increase in arterial sprouting and VSMC migration in stroke after Niaspan treatment is partially attributed to the increased expression of TACE in the ischemic brain and cerebral arteries (Chen et al., 2009).

High-density lipoprotein, in addition to promoting arteriogenesis, also up-regulates angiogenesis post-ischemic stroke (Chen et al., 2007). Recent findings in human and *in vitro* cell culture show that niacin impedes apolipoprotein A-1 (APO A-1) hepatic catabolism, thus prolonging HDL half-life (Jin et al., 1997; Kamanna and Kashyap, 2008). HDL promotes endothelial progenitor cell incorporation, endothelial cell migration, and re-endothelialization, all of which are mediated by eNOS and phosphoinositide 3-kinase (PI3k)/Akt kinase activation (Shehadah et al., 2010b). Treatments that combine low doses of Niaspan and tPA administered 4 h after stroke significantly improved functional outcome, reduced lesion volume, decreased expression of TLR4 and TNF-α, and decreased apoptosis in MCAo rats (Shehadah et al., 2011). Combination of Niaspan with Simvastatin helped improve overall functional outcome significantly and decreased axonal damage and density (Shehadah et al., 2010a).

## Conclusion

Primary physiological mediators of neurorecovery post-stroke, including angiogenesis, arteriogenesis, neurogenesis, and white matter remodeling, have been described in this review. In addition, select cell-based therapies (HUCBCs and BMSCs), and an example of a restorative pharmacological agent, Niaspan, which increases HDL, which amplifies these restorative processes as restorative treatments for stroke have been discussed. Elucidating the underlying mechanisms of cell-based and pharmacological restorative therapies is of primary interest and crucial for translation of treatments to clinical use. miRNAs are major molecular regulators and appear to have pivotal roles in cell-based and possibly pharmacological restorative therapies for stroke. Clarification of their roles in mediating neurorecovery post-stroke, warrant further investigation.

## Conflict of Interest Statement

The authors declare that the research was conducted in the absence of any commercial or financial relationships that could be construed as a potential conflict of interest.

## References

[B1] Allen C. M. (1984). Predicting the outcome of acute stroke: a prognostic score. J. Neurol. Neurosurg. Psychiatry 47, 475–480 10.1136/jnnp.47.5.475 6736978 PMC1027822

[B2] Almici C. Carlo-Stella C. Wagner J. E. Rizzoli V. (1995). Umbilical cord blood as a source of hematopoietic stem cells: from research to clinical application. Haematologica 80, 473–479 8566892

[B3] Andrews E. M. Tsai S. Y. Johnson S. C. Farrer J. R. Wagner J. P. Kopen G. C. (2008). Human adult bone marrow-derived somatic cell therapy results in functional recovery and axonal plasticity following stroke in the rat. Exp. Neurol. 211, 588–592 10.1016/j.expneurol.2008.02.027 18440506 PMC3932708

[B4] Arenillas J. F. Sobrino T. Castillo J. Davalos A. (2007). The role of angiogenesis in damage and recovery from ischemic stroke. Curr. Treat. Options Cardiovasc. Med. 9, 205–212 10.1007/s11936-007-0014-5 17601384

[B5] Arien-Zakay H. Lecht S. Nagler A. Lazarovici P. (2011). Neuroprotection by human umbilical cord blood-derived progenitors in ischemic brain injuries. Arch. Ital. Biol. 149, 233–245 10.4449/aib.v149i2.1370 21701995

[B6] Back S. A. Han B. H. Luo N. L. Chricton C. A. Xanthoudakis S. Tam J. (2002). Selective vulnerability of late oligodendrocyte progenitors to hypoxia-ischemia. J. Neurosci. 22, 455–463 11784790 10.1523/JNEUROSCI.22-02-00455.2002 PMC6758669

[B7] Bang O. Y. Lee J. S. Lee P. H. Lee G. (2005). Autologous mesenchymal stem cell transplantation in stroke patients. Ann. Neurol. 57, 874–882 10.1002/ana.20501 15929052

[B8] Bao X. Wei J. Feng M. Lu S. Li G. Dou W. (2011). Transplantation of human bone marrow-derived mesenchymal stem cells promotes behavioral recovery and endogenous neurogenesis after cerebral ischemia in rats. Brain Res. 7, 103–113 10.1016/j.brainres.2010.10.063 20977892

[B9] Berezovskaya O. Maysinger D. Fedoroff S. (1995). The hematopoietic cytokine, colony-stimulating factor 1, is also a growth factor in the CNS: congenital absence of CSF-1 in mice results in abnormal microglial response and increased neuron vulnerability to injury. Int. J. Dev. Neurosci. 13, 285–299 10.1016/0736-5748(95)00013-7 7572282

[B10] Bianco P. Gehron Robey P. (2000). Marrow stromal stem cells. J. Clin. Invest. 105, 1663–1668 10.1172/JCI10413 10862779 PMC378520

[B11] Buschmann I. Schaper W. (1999). Arteriogenesis versus angiogenesis: two mechanisms of vessel growth. News Physiol. Sci. 14, 121–125 11390835 10.1152/physiologyonline.1999.14.3.121

[B12] Buschmann I. Schaper W. (2000). The pathophysiology of the collateral circulation (arteriogenesis). J. Pathol. 190, 338–342 10.1002/(SICI)1096-9896(200002)190:3<338::AID-PATH594>3.0.CO,2-7 10685067

[B13] Cairns K. Finklestein S. P. (2003). Growth factors and stem cells as treatments for stroke recovery. Phys. Med. Rehabil. Clin. N. Am. 14, S135–S142 10.1016/S1047-9651(02)00059-1 12625643

[B14] Carmeliet P. (2000). Mechanisms of angiogenesis and arteriogenesis. Nat. Med. 6, 389–395 10.1038/80430 10742145

[B15] Carmichael S. T. (2003). Plasticity of cortical projections after stroke. Neuroscientist. 9, 64–75 10.1177/1073858402239592 12580341

[B16] Carmichael S. T. Wei L. Rovainen C. M. Woolsey T. A. (2001). New patterns of intracortical projections after focal cortical stroke. Neurobiol. Dis. 8, 910–922 10.1006/nbdi.2001.0425 11592858

[B17] Carpenter C. R. Keim S. M. Milne W. K. Meurer W. J. Barsan W. G. (2011). Thrombolytic therapy for acute ischemic stroke beyond three hours. J. Emerg. Med. 40, 82–92 10.1016/j.jemermed.2010.05.009 20576390 PMC3217216

[B18] Chang E. H. Rigotti A. Huerta P. T. (2009). Age-related influence of the HDL receptor SR-BI on synaptic plasticity and cognition. Neurobiol. Aging 30, 407–419 10.1016/j.neurobiolaging.2007.07.006 17719144 PMC2665297

[B19] Chang S. J. Weng S. L. Hsieh J. Y. Wang T. Y. Chang M. D. Wang H. W. (2011). MicroRNA-34a modulates genes involved in cellular motility and oxidative phosphorylation in neural precursors derived from human umbilical cord mesenchymal stem cells. BMC Med. Genomics 4:65 10.1186/1755-8794-4-65 21923954 PMC3195087

[B20] Chen C. T. Foo N. H. Liu W. S. Chen S. H. (2008). Infusion of human umbilical cord blood cells ameliorates hind limb dysfunction in experimental spinal cord injury through anti-inflammatory, vasculogenic and neurotrophic mechanisms. Pediatr. Neonatol. 49, 77–83 10.1016/S1875-9572(08)60017-0 18947003

[B21] Chen J. Chopp M. (2006). Neurorestorative treatment of stroke: cell and pharmacological approaches. NeuroRx 3, 466–473 10.1016/j.nurx.2006.07.007 17012060 PMC2790719

[B22] Chen J. Cui X. Zacharek A. Cui Y. Roberts C. Chopp M. (2011). White matter damage and the effect of matrix metalloproteinases in type 2 diabetic mice after stroke. Stroke 42, 445–452 10.1161/STROKEAHA.110.596486 21193743 PMC3108495

[B23] Chen J. Cui X. Zacharek A. Ding G. L. Shehadah A. Jiang Q. (2009). Niaspan treatment increases tumor necrosis factor-alpha-converting enzyme and promotes arteriogenesis after stroke. J. Cereb. Blood Flow Metab. 29, 911–920 10.1038/jcbfm.2009.11 19223914 PMC2782460

[B24] Chen J. Cui X. Zacharek A. Jiang H. Roberts C. Zhang C. (2007). Niaspan increases angiogenesis and improves functional recovery after stroke. Ann. Neurol. 62, 49–58 10.1002/ana.21160 17557352

[B25] Chen J. Li Y. Katakowski M. Chen X. Wang L. Lu D. (2003a). Intravenous bone marrow stromal cell therapy reduces apoptosis and promotes endogenous cell proliferation after stroke in female rat. J. Neurosci. Res. 73, 778–786 10.1002/jnr.10691 12949903

[B26] Chen J. Li Y. Wang L. Zhang Z. Lu D. Lu M. (2001a). Therapeutic benefit of intravenous administration of bone marrow stromal cells after cerebral ischemia in rats. Stroke 32, 1005–1011 10.1161/01.STR.32.4.1005 11283404

[B27] Chen J. Sanberg P. R. Li Y. Wang L. Lu M. Willing A. E. (2001b). Intravenous administration of human umbilical cord blood reduces behavioral deficits after stroke in rats. Stroke 32, 2682–2688 10.1161/hs1101.098367 11692034

[B28] Chen J. Zacharek A. Cui X. Shehadah A. Jiang H. Roberts C. (2010). Treatment of stroke with a synthetic liver X receptor agonist, TO901317, promotes synaptic plasticity and axonal regeneration in mice. J. Cereb. Blood Flow Metab. 30, 102–109 10.1038/jcbfm.2009.187 19724285 PMC2804900

[B29] Chen J. Zacharek A. Li Y. Li A. Wang L. Katakowski M. (2006). N-cadherin mediates nitric oxide-induced neurogenesis in young and retired breeder neurospheres. Neuroscience 140, 377–388 10.1016/j.neuroscience.2006.02.064 16580782 PMC2791333

[B30] Chen J. Zhang Z. G. Li Y. Wang L. Xu Y. X. Gautam S. C. (2003b). Intravenous administration of human bone marrow stromal cells induces angiogenesis in the ischemic boundary zone after stroke in rats. Circ. Res. 92, 692–699 10.1161/01.RES.0000063425.51108.8D 12609969

[B31] Chen J. J. Zhou S. H. (2011). Mesenchymal stem cells overexpressing MiR-126 enhance ischemic angiogenesis via the AKT/ERK-related pathway. Cardiol. J. 18, 675–681 10.5603/CJ.2011.0032 22113756

[B32] Chen S. H. Wang J. J. Chen C. H. Chang H. K. Lin M. T. Chang F. M. (2013). Umbilical cord blood-derived CD34+ cells improve outcomes of traumatic brain injury in rats by stimulating angiogenesis and neurogenesis. Cell Transplant. 12, 12 23582375 10.3727/096368913X667006

[B33] Chen X. Katakowski M. Li Y. Lu D. Wang L. Zhang L. (2002a). Human bone marrow stromal cell cultures conditioned by traumatic brain tissue extracts: growth factor production. J. Neurosci. Res. 69, 687–691 10.1002/jnr.10334 12210835

[B34] Chen X. Li Y. Wang L. Katakowski M. Zhang L. Chen J. (2002b). Ischemic rat brain extracts induce human marrow stromal cell growth factor production. Neuropathology 22, 275–279 10.1046/j.1440-1789.2002.00450.x 12564767

[B35] Chopp M. Li Y. Zhang Z. G. (2009). Mechanisms underlying improved recovery of neurological function after stroke in the rodent after treatment with neurorestorative cell-based therapies. Stroke 40, S143–S145 10.1161/STROKEAHA.108.533141 19064763 PMC2854491

[B36] Christoforidis G. A. Mohammad Y. Kehagias D. Avutu B. Slivka A. P. (2005). Angiographic assessment of pial collaterals as a prognostic indicator following intra-arterial thrombolysis for acute ischemic stroke. AJNR Am. J. Neuroradiol. 26, 1789–1797 16091531 PMC7975179

[B37] Collino F. Bruno S. Deregibus M. C. Tetta C. Camussi G. (2011). “Chapter fourteen – microRNAs and mesenchymal stem cells,” in Vitamins & Hormones, ed. Gerald L. (Academic Press), 291–320 10.1016/B978-0-12-386015-6.00033-0 22127248

[B38] Cramer S. C. Chopp M. (2000). Recovery recapitulates ontogeny. Trends Neurosci. 23, 265–271 10.1016/S0166-2236(00)01562-9 10838596

[B39] Cronin C. A. (2010). Intravenous tissue plasminogen activator for stroke: a review of the ECASS III results in relation to prior clinical trials. J. Emerg. Med. 38, 99–105 10.1016/j.jemermed.2009.08.004 19765940

[B40] Cui X. Chopp M. Zacharek A. Roberts C. Buller B. Ion M. (2010). Niacin treatment of stroke increases synaptic plasticity and axon growth in rats. Stroke 41, 2044–2049 10.1161/STROKEAHA.110.589333 20671245 PMC2932778

[B41] Cui X. Chopp M. Zacharek A. Roberts C. Lu M. Savant-Bhonsale S. (2009). Chemokine, vascular and therapeutic effects of combination Simvastatin and BMSC treatment of stroke. Neurobiol. Dis. 36, 35–41 10.1016/j.nbd.2009.06.012 19591934 PMC2748847

[B42] Dancause N. Barbay S. Frost S. B. Plautz E. J. Chen D. Zoubina E. V. (2005). Extensive cortical rewiring after brain injury. J. Neurosci. 25, 10167–10179 10.1523/JNEUROSCI.3256-05.2005 16267224 PMC6725801

[B43] Ding J. Cheng Y. Gao S. Chen J. (2011). Effects of nerve growth factor and Noggin-modified bone marrow stromal cells on stroke in rats. J. Neurosci. Res. 89, 222–230 10.1002/jnr.22535 21162129

[B44] Dor Y. Keshet E. (1997). Ischemia-driven angiogenesis. Trends Cardiovasc. Med. 7, 289–294 10.1016/S1050-1738(97)00091-1 21235898

[B45] Erdo F. Buschmann I. R. (2007). Arteriogenesis: a new strategy of therapeutic intervention in chronic arterial disorders. Cellular mechanism and experimental models. Orv. Hetil. 148, 633–642 17403636 10.1556/OH.2007.27916

[B46] Feng Y. Wang Y. Pfister F. Hillebrands J. L. Deutsch U. Hammes H. P. (2009). Decreased hypoxia-induced neovascularization in angiopoietin-2 heterozygous knockout mouse through reduced MMP activity. Cell. Physiol. Biochem. 23, 277–284 10.1159/000218174 19471095

[B47] Folkman J. D’Amore P. A. (1996). Blood vessel formation: what is its molecular basis? Cell 87, 1153–1155 8980221 10.1016/s0092-8674(00)81810-3

[B48] Friedenstein A. J. Chailakhyan R. K. Gerasimov U. V. (1987). Bone marrow osteogenic stem cells: in vitro cultivation and transplantation in diffusion chambers. Cell Tissue Kinet. 20, 263–272 3690622 10.1111/j.1365-2184.1987.tb01309.x

[B49] Friedenstein A. J. Petrakova K. V. Kurolesova A. I. Frolova G. P. (1968). Heterotopic of bone marrow. Analysis of precursor cells for osteogenic and hematopoietic tissues. Transplantation 6, 230–247 10.1097/00007890-196803000-00009 5654088

[B50] Gensert J. M. Goldman J. E. (1997). Endogenous progenitors remyelinate demyelinated axons in the adult CNS. Neuron 19, 197–203 10.1016/S0896-6273(00)80359-1 9247275

[B51] Granger C. V. Hamilton B. B. Fiedler R. C. (1992). Discharge outcome after stroke rehabilitation. Stroke 23, 978–982 1615548 10.1161/01.str.23.7.978

[B52] Greenberg D. A. (1998). Angiogenesis and stroke. Drug News Perspect. 11, 265–270 10.1358/dnp.1998.11.5.657287 15616645

[B53] Grotta J. C. Jacobs T. P. Koroshetz W. J. Moskowitz M. A. (2008). Stroke program review group: an interim report. Stroke 39, 1364–1370 10.1161/STROKEAHA.107.510776 18309142

[B54] Gutierrez-Fernandez M. Rodriguez-Frutos B. Ramos-Cejudo J. Teresa Vallejo-Cremades M. Fuentes B. Cerdan S. (2013). Effects of intravenous administration of allogenic bone marrow- and adipose tissue-derived mesenchymal stem cells on functional recovery and brain repair markers in experimental ischemic stroke. Stem Cell Res Ther. 4, 11 10.1186/scrt159 23356495 PMC3706777

[B55] Hardy S. A. Maltman D. J. Przyborski S. A. (2008). Mesenchymal stem cells as mediators of neural differentiation. Curr. Stem Cell Res. Ther. 3, 43–52 10.2174/157488808783489471 18220922

[B56] Henning R. J. Abu-Ali H. Balis J. U. Morgan M. B. Willing A. E. Sanberg P. R. (2004). Human umbilical cord blood mononuclear cells for the treatment of acute myocardial infarction. Cell Transplant. 13, 729–739 10.3727/000000004783983477 15690974

[B57] Hermann D. M. Chopp M. (2012). Promoting brain remodelling and plasticity for stroke recovery: therapeutic promise and potential pitfalls of clinical translation. Lancet Neurol. 11, 369–380 10.1016/S1474-4422(12)70039-X 22441198 PMC3964179

[B58] Hoefer I. E. van Royen N. Rectenwald J. E. Bray E. J. Abouhamze Z. Moldawer L. L. (2002). Direct evidence for tumor necrosis factor-alpha signaling in arteriogenesis. Circulation 105, 1639–1641 10.1161/01.CIR.0000014987.32865.8E 11940540

[B59] Hou S. T. Jiang S. X. Smith R. A. (2008). Permissive and repulsive cues and signalling pathways of axonal outgrowth and regeneration. Int. Rev. Cell Mol. Biol. 267, 125–181 10.1016/S1937-6448(08)00603-5 18544498

[B60] Hu C. H. Wu G. F. Wang X. Q. Yang Y. H. Du Z. M. He X. H. (2006). Transplanted human umbilical cord blood mononuclear cells improve left ventricular function through angiogenesis in myocardial infarction. Chin. Med. J. 119, 1499–1506 16996002

[B61] Huang F. Zhu X. Hu X. Q. Fang Z. F. Tang L. Lu X. L. (2013a). Mesenchymal stem cells modified with miR-126 release angiogenic factors and activate Notch ligand Delta-like-4, enhancing ischemic angiogenesis and cell survival. Int. J. Mol. Med. 31, 484–492 10.3892/ijmm.2012.1200 23229021

[B62] Huang W. Mo X. Qin C. Zheng J. Liang Z. Zhang C. (2013b). Transplantation of differentiated bone marrow stromal cells promotes motor functional recovery in rats with stroke. Neurol. Res. 35, 320–328 10.1179/1743132812Y.0000000151 23485057

[B63] Hurtado O. Pradillo J. M. Alonso-Escolano D. Lorenzo P. Sobrino T. Castillo J. (2006). Neurorepair versus neuroprotection in stroke. Cerebrovasc. Dis. 21(Suppl. 2), 54–63 10.1159/000091704 16651815

[B64] Iwai M. Stetler R. A. Xing J. Hu X. Gao Y. Zhang W. (2010). Enhanced oligodendrogenesis and recovery of neurological function by erythropoietin after neonatal hypoxic/ischemic brain injury. Stroke 41, 1032–1037 10.1161/STROKEAHA.109.570325 20360553 PMC2919308

[B65] Jiang Y. Zhu W. Zhu J. Wu L. Xu G. Liu X. (2012). Feasibility of delivering mesenchymal stem cells via catheter to the proximal end of lesion artery in patients with stroke in the territory of middle cerebral artery. Cell Transplant. 1, 1 10.3727/096368912X658818 23127560

[B66] Jin F.-Y. Kamanna V. S. Kashyap M. L. (1997). Niacin decreases removal of high-density lipoprotein apolipoprotein A-I but not cholesterol ester by Hep G2 cells: implication for reverse cholesterol transport. Arterioscler. Thromb. Vasc. Biol. 17, 2020–2028 10.1161/01.ATV.17.10.2020 9351367

[B67] Juranek J. K. Geddis M. S. Song F. Zhang J. Garcia J. Rosario R. (2013). RAGE deficiency improves postinjury sciatic nerve regeneration in type 1 diabetic mice. Diabetes 62, 931–943 10.2337/db12-0632 23172920 PMC3581233

[B68] Kamanna V. S. Kashyap M. L. (2008). Mechanism of action of niacin. Am. J. Cardiol. 101, 029 10.1016/j.amjcard.2008.02.029 18375237

[B69] Karasinska J. M. Rinninger F. Lutjohann D. Ruddle P. Franciosi S. Kruit J. K. (2009). Specific loss of brain ABCA1 increases brain cholesterol uptake and influences neuronal structure and function. J. Neurosci. 29, 3579–3589 10.1523/JNEUROSCI.4741-08.2009 19295162 PMC2678874

[B70] Katakowski M. Buller B. Zheng X. Lu Y. Rogers T. Osobamiro O. (2013). Exosomes from marrow stromal cells expressing miR-146b inhibit glioma growth. Cancer Lett. 335, 201–204 10.1016/j.canlet.2013.02.019 23419525 PMC3665755

[B71] Katsuda T. Kosaka N. Takeshita F. Ochiya T. (2013). The therapeutic potential of mesenchymal stem cell-derived extracellular vesicles. Proteomics 13, 1637–1653 10.1002/pmic.201200373 23335344

[B72] Katzan I. L. Hammer M. D. Hixson E. D. Furlan A. J. Abou-Chebl A. Nadzam D. M. (2004). Utilization of intravenous tissue plasminogen activator for acute ischemic stroke. Arch. Neurol. 61, 346–350 10.1001/archneur.61.3.346 15023810

[B73] Kinnaird T. Stabile E. Burnett M. S. Lee C. W. Barr S. Fuchs S. (2004). Marrow-derived stromal cells express genes encoding a broad spectrum of arteriogenic cytokines and promote in vitro and in vivo arteriogenesis through paracrine mechanisms. Circ. Res. 94, 678–685 10.1161/01.RES.0000118601.37875.AC 14739163

[B74] Kobayashi T. Ahlenius H. Thored P. Kobayashi R. Kokaia Z. Lindvall O. (2006). Intracerebral infusion of glial cell line-derived neurotrophic factor promotes striatal neurogenesis after stroke in adult rats. Stroke 37, 2361–2367 10.1161/01.STR.0000236025.44089.e1 16873711

[B75] Kozlowska H. Jablonka J. Janowski M. Jurga M. Kossut M. Domanska-Janik K. (2007). Transplantation of a novel human cord blood-derived neural-like stem cell line in a rat model of cortical infarct. Stem Cells Dev. 16, 481–488 10.1089/scd.2007.9993 17610378

[B76] Krupinski J. Kaluza J. Kumar P. Kumar S. Wang J. M. (1994). Role of angiogenesis in patients with cerebral ischemic stroke. Stroke 25, 1794–1798 10.1161/01.STR.25.9.1794 7521076

[B77] Laine S. K. Alm J. J. Virtanen S. P. Aro H. T. Laitala-Leinonen T. K. (2012). MicroRNAs miR-96, miR-124, and miR-199a regulate gene expression in human bone marrow-derived mesenchymal stem cells. J. Cell. Biochem. 113, 2687–2695 10.1002/jcb.24144 22441842

[B78] Landers M. (2004). Treatment-induced neuroplasticity following focal injury to the motor cortex. Int. J. Rehabil. Res. 27, 1–5 10.1097/00004356-200403000-00001 15097164

[B79] Lee J. S. Hong J. M. Moon G. J. Lee P. H. Ahn Y. H. Bang O. Y. (2010). A long-term follow-up study of intravenous autologous mesenchymal stem cell transplantation in patients with ischemic stroke. Stem Cells 28, 1099–1106 10.1002/stem.430 20506226

[B80] Leonardo C. C. Hall A. A. Collier L. A. Ajmo C. T. Jr. Willing A. E. Pennypacker K. R. (2010). Human umbilical cord blood cell therapy blocks the morphological change and recruitment of CD11b-expressing, isolectin-binding proinflammatory cells after middle cerebral artery occlusion. J. Neurosci. Res. 88, 1213–1222 10.1002/jnr.22306 19998484 PMC2830323

[B81] Li Y. Chen J. Chen X. G. Wang L. Gautam S. C. Xu Y. X. (2002). Human marrow stromal cell therapy for stroke in rat: neurotrophins and functional recovery. Neurology 59, 514–523 10.1212/WNL.59.4.514 12196642

[B82] Li Y. Chen J. Chopp M. (2001a). Adult bone marrow transplantation after stroke in adult rats. Cell Transplant. 10, 31–40 11294470

[B83] Li Y. Chen J. Wang L. Lu M. Chopp M. (2001b). Treatment of stroke in rat with intracarotid administration of marrow stromal cells. Neurology 56, 1666–1672 10.1212/WNL.56.12.1666 11425931

[B84] Liebeskind D. S. (2005). Neuroprotection from the collateral perspective. Idrugs 8, 222–228 15772894

[B85] Lim P. K. Patel S. A. Gregory L. A. Rameshwar P. (2010). Neurogenesis: role for microRNAs and mesenchymal stem cells in pathological states. Curr. Med. Chem. 17, 2159–2167 10.2174/092986710791299894 20423304

[B86] Liu N. Deguchi K. Yamashita T. Liu W. Ikeda Y. Abe K. (2012). Intracerebral transplantation of bone marrow stromal cells ameliorates tissue plasminogen activator-induced brain damage after cerebral ischemia in mice detected by in vivo and ex vivo optical imaging. J. Neurosci. Res. 90, 2086–2093 10.1002/jnr.23104 22791305

[B87] Liu X. Ye R. Yan T. Yu S. P. Wei L. Xu G. (2014). Cell based therapies for ischemic stroke: from basic science to bedside. Prog. Neurobiol. 115, 92–115 10.1016/j.pneurobio.2013.11.007 24333397 PMC4038267

[B88] Liu Z. Li Y. Zhang Z. G. Cui X. Cui Y. Lu M. (2010). Bone marrow stromal cells enhance inter- and intra-cortical axonal connections after ischemic stroke in adult rats. J. Cereb. Blood Flow Metab. 30, 1288–1295 10.1038/jcbfm.2010.8 20125183 PMC2896436

[B89] Liu Z. Zhang R. L. Li Y. Cui Y. Chopp M. (2009). Remodeling of the corticospinal innervation and spontaneous behavioral recovery after ischemic stroke in adult mice. Stroke 40, 2546–2551 10.1161/STROKEAHA.109.547265 19478220 PMC2704262

[B90] Lu D. Sanberg P. R. Mahmood A. Li Y. Wang L. Sanchez-Ramos J. (2002). Intravenous administration of human umbilical cord blood reduces neurological deficit in the rat after traumatic brain injury. Cell Transplant. 11, 275–281 12075993

[B91] Lutsenko S. V. Kiselev S. M. Severin S. E. (2003). Molecular mechanisms of tumor angiogenesis. Biochemistry Mosc. 68, 286–300 12733970 10.1023/a:1023002216413

[B92] Madri J. A. (2009). Modeling the neurovascular niche: implications for recovery from CNS injury. J. Physiol. Pharmacol. 4, 95–104 20083857

[B93] Malgieri A. Kantzari E. Patrizi M. P. Gambardella S. (2010). Bone marrow and umbilical cord blood human mesenchymal stem cells: state of the art. Int. J. Clin. Exp. Med. 3, 248–269 21072260 PMC2971538

[B94] Matsuda-Hashii Y. Takai K. Ohta H. Fujisaki H. Tokimasa S. Osugi Y. (2004). Hepatocyte growth factor plays roles in the induction and autocrine maintenance of bone marrow stromal cell IL-11, SDF-1 alpha, and stem cell factor. Exp. Hematol. 32, 955–961 15504551 10.1016/j.exphem.2004.06.012

[B95] Matsui M. Tabata Y. (2012). Enhanced angiogenesis by multiple release of platelet-rich plasma contents and basic fibroblast growth factor from gelatin hydrogels. Acta Biomater. 8, 1792–1801 10.1016/j.actbio.2012.01.016 22293581

[B96] McTigue D. M. Tripathi R. B. (2008). The life, death, and replacement of oligodendrocytes in the adult CNS. J. Neurochem. 107, 1–19 10.1111/j.1471-4159.2008.05570.x 18643793

[B97] Mehler M. F. Rozental R. Dougherty M. Spray D. C. Kessler J. A. (1993). Cytokine regulation of neuronal differentiation of hippocampal progenitor cells. Nature 362, 62–65 10.1038/362062a0 8383296

[B98] Miller A. M. Gilchrist D. S. Nijjar J. Araldi E. Ramirez C. M. Lavery C. A. (2013). MiR-155 has a protective role in the development of non-alcoholic hepatosteatosis in mice. PLoS ONE 8:e72324 10.1371/journal.pone.0072324 23991091 PMC3749101

[B99] Modo M. Rezaie P. Heuschling P. Patel S. Male D. K. Hodges H. (2002a). Transplantation of neural stem cells in a rat model of stroke: assessment of short-term graft survival and acute host immunological response. Brain Res. 958, 70–82 10.1016/S0006-8993(02)03463-7 12468031

[B100] Modo M. Stroemer R. P. Tang E. Patel S. Hodges H. (2002b). Effects of implantation site of stem cell grafts on behavioral recovery from stroke damage. Stroke 33, 2270–2278 10.1161/01.STR.0000027693.50675.C5 12215598

[B101] Moniche F. Gonzalez A. Gonzalez-Marcos J. R. Carmona M. Pinero P. Espigado I. (2012). Intra-arterial bone marrow mononuclear cells in ischemic stroke: a pilot clinical trial. Stroke 43, 2242–2244 10.1161/STROKEAHA.112.659409 22764211

[B102] Morgado J. M. Pratas R. Laranjeira P. Henriques A. Crespo I. Regateiro F. (2008). The phenotypical and functional characteristics of cord blood monocytes and CD14(-/low)/CD16(+) dendritic cells can be relevant to the development of cellular immune responses after transplantation. Transpl. Immunol. 19, 55–63 10.1016/j.trim.2007.11.002 18346638

[B103] Munoz J. R. Stoutenger B. R. Robinson A. P. Spees J. L. Prockop D. J. (2005). Human stem/progenitor cells from bone marrow promote neurogenesis of endogenous neural stem cells in the hippocampus of mice. Proc. Natl. Acad. Sci. U.S.A. 102, 18171–18176 10.1073/pnas.0508945102 16330757 PMC1312406

[B104] Nakahata T. Ogawa M. (1982). Hemopoietic colony-forming cells in umbilical cord blood with extensive capability to generate mono- and multi-potential hemopoietic progenitors. J. Clin. Invest. 70, 1324–1328 10.1172/JCI110734 7174797 PMC370352

[B105] Navarro-Sobrino M. Rosell A. Hernandez-Guillamon M. Penalba A. Boada C. Domingues-Montanari S. (2011). A large screening of angiogenesis biomarkers and their association with neurological outcome after ischemic stroke. Atherosclerosis 216, 205–211 10.1016/j.atherosclerosis.2011.01.030 21324462

[B106] Neuhoff S. Moers J. Rieks M. Grunwald T. Jensen A. Dermietzel R. (2007). Proliferation, differentiation, and cytokine secretion of human umbilical cord blood-derived mononuclear cells in vitro. Exp. Hematol. 35, 1119–1131 10.1016/j.exphem.2007.03.019 17588481

[B107] Newcomb J. D. Ajmo C. T. Jr. Sanberg C. D. Sanberg P. R. Pennypacker K. R. Willing A. E. (2006). Timing of cord blood treatment after experimental stroke determines therapeutic efficacy. Cell Transplant. 15, 213–223 10.3727/000000006783982043 16719056

[B108] Nikolic I. Plate K. H. Schmidt M. H. (2010). EGFL7 meets miRNA-126: an angiogenesis alliance. J. Angiogenes. Res. 2, 9 10.1186/2040-2384-2-9 20529320 PMC2901201

[B109] Nikolic W. V. Hou H. Town T. Zhu Y. Giunta B. Sanberg C. D. (2008). Peripherally administered human umbilical cord blood cells reduce parenchymal and vascular beta-amyloid deposits in Alzheimer mice. Stem Cells Dev. 17, 423–439 10.1089/scd.2008.0018 18366296 PMC2649688

[B110] NINDS. (2012). Final Report of the Stroke Progress Review Group – January 2012.

[B111] Nystedt J. Makinen S. Laine J. Jolkkonen J. (2006). Human cord blood CD34+ cells and behavioral recovery following focal cerebral ischemia in rats. Acta Neurobiol. Exp. 66, 293–300 17265691 10.55782/ane-2006-1618

[B112] Ohab J. J. Fleming S. Blesch A. Carmichael S. T. (2006). A neurovascular niche for neurogenesis after stroke. J. Neurosci. 26, 13007–13016 10.1523/JNEUROSCI.4323-06.2006 17167090 PMC6674957

[B113] Parent J. M. Vexler Z. S. Gong C. Derugin N. Ferriero D. M. (2002). Rat forebrain neurogenesis and striatal neuron replacement after focal stroke. Ann. Neurol. 52, 802–813 10.1002/ana.10393 12447935

[B114] Patan S. (2004). Vasculogenesis and angiogenesis. Cancer Treat. Res. 117, 3–32 15015550 10.1007/978-1-4419-8871-3_1

[B115] Plate K. H. (1999). Mechanisms of angiogenesis in the brain. J. Neuropathol. Exp. Neurol. 58, 313–320 10.1097/00005072-199904000-00001 10218626

[B116] Pratt P. F. Medhora M. Harder D. R. (2004). Mechanisms regulating cerebral blood flow as therapeutic targets. Curr. Opin. Investig. Drugs 5, 952–956 15503650

[B117] Renner O. Tsimpas A. Kostin S. Valable S. Petit E. Schaper W. (2003). Time- and cell type-specific induction of platelet-derived growth factor receptor-beta during cerebral ischemia. Brain Res. Mol. Brain Res. 113, 44–51 12750005 10.1016/s0169-328x(03)00085-8

[B118] Riess P. Zhang C. Saatman K. E. Laurer H. L. Longhi L. G. Raghupathi R. (2002). Transplanted neural stem cells survive, differentiate, and improve neurological motor function after experimental traumatic brain injury. Neurosurgery 51, 1043–1052 10.1097/00006123-200210000-00035 12234415

[B119] Risau W. (1997). Mechanisms of angiogenesis. Nature 386, 671–674 10.1038/386671a0 9109485

[B120] Rowe D. D. Leonardo C. C. Hall A. A. Shahaduzzaman M. D. Collier L. A. Willing A. E. (2010). Cord blood administration induces oligodendrocyte survival through alterations in gene expression. Brain Res. 17, 172–188 10.1016/j.brainres.2010.09.078 20883670 PMC2993822

[B121] Rowe D. D. Leonardo C. C. Recio J. A. Collier L. A. Willing A. E. Pennypacker K. R. (2012). Human umbilical cord blood cells protect oligodendrocytes from brain ischemia through Akt signal transduction. J. Biol. Chem. 287, 4177–4187 10.1074/jbc.M111.296434 22158864 PMC3281708

[B122] Savitz S. L. Malhotra S. Gupta G. Rosenbaum D. M. (2003). Cell transplants offer promise for stroke recovery. J. Cardiovasc. Nurs. 18, 57–61 10.1097/00005082-200301000-00009 12537091

[B123] Schaper W. Buschmann I. (1999). Arteriogenesis, the good and bad of it. Eur. Heart J. 20, 1297–1299 10.1053/euhj.1999.1686 10462463

[B124] Scholz D. Cai W. J. Schaper W. (2001). Arteriogenesis, a new concept of vascular adaptation in occlusive disease. Angiogenesis 4, 247–257 10.1023/A:1016094004084 12197469

[B125] Schwammenthal Y. Tsabari R. Bakon M. Orion D. Merzeliak O. Tanne D. (2006). Thrombolysis in acute stroke. Isr. Med. Assoc. J. 8, 784–787 17180831

[B126] Seetharam D. Mineo C. Gormley A. K. Gibson L. L. Vongpatanasin W. Chambliss K. L. (2006). High-density lipoprotein promotes endothelial cell migration and reendothelialization via scavenger receptor-B type I. Circ. Res. 98, 63–72 10.1161/01.RES.0000199272.59432.5b 16339487

[B127] Sen C. K. (2011). MicroRNAs as new maestro conducting the expanding symphony orchestra of regenerative and reparative medicine. Physiol. Genomics 43, 517–520 10.1152/physiolgenomics.00037.2011 21467158 PMC3110892

[B128] Shehadah A. Chen J. Cui X. Roberts C. Lu M. Chopp M. (2010a). Combination treatment of experimental stroke with Niaspan and Simvastatin, reduces axonal damage and improves functional outcome. J. Neurol. Sci. 294, 107–111 10.1016/j.jns.2010.03.020 20451219 PMC2885546

[B129] Shehadah A. Chen J. Cui Y. Zhang L. Roberts C. Lu M. (2011). Combination treatment with low-dose Niaspan and tissue plasminogen activator provides neuroprotection after embolic stroke in rats. J. Neurol. Sci. 309, 96–101 10.1016/j.jns.2011.07.008 21802695 PMC3166443

[B130] Shehadah A. Chen J. Zacharek A. Cui Y. Ion M. Roberts C. (2010b). Niaspan treatment induces neuroprotection after stroke. Neurobiol. Dis. 40, 277–283 10.1016/j.nbd.2010.05.034 20554037 PMC2926170

[B131] Shen L. H. Li Y. Chen J. Zacharek A. Gao Q. Kapke A. (2007). Therapeutic benefit of bone marrow stromal cells administered 1 month after stroke. J. Cereb. Blood Flow Metab. 27, 6–13 10.1038/sj.jcbfm.9600311 16596121

[B132] Shen L. H. Li Y. Chen J. Zhang J. Vanguri P. Borneman J. (2006). Intracarotid transplantation of bone marrow stromal cells increases axon-myelin remodeling after stroke. Neuroscience 137, 393–399 10.1016/j.neuroscience.2005.08.092 16298076

[B133] Shichinohe H. Kuroda S. Maruichi K. Osanai T. Sugiyama T. Chiba Y. (2010). Bone marrow stromal cells and bone marrow-derived mononuclear cells: which are suitable as cell source of transplantation for mice infarct brain? Neuropathology 30, 113–122 10.1111/j.1440-1789.2009.01050.x 19737360

[B134] Skihar V. Silva C. Chojnacki A. Doring A. Stallcup W. B. Weiss S. (2009). Promoting oligodendrogenesis and myelin repair using the multiple sclerosis medication glatiramer acetate. Proc. Natl. Acad. Sci. U.S.A. 106, 17992–17997 10.1073/pnas.0909607106 19815532 PMC2758287

[B135] Stroemer R. P. Kent T. A. Hulsebosch C. E. (1995). Neocortical neural sprouting, synaptogenesis, and behavioral recovery after neocortical infarction in rats. Stroke 26, 2135–2144 10.1161/01.STR.26.11.2135 7482662

[B136] Suarez-Monteagudo C. Hernandez-Ramirez P. Alvarez-Gonzalez L. Garcia-Maeso I. de la Cuetara-Bernal K. Castillo-Diaz L. (2009). Autologous bone marrow stem cell neurotransplantation in stroke patients. An open study. Restor. Neurol. Neurosci. 27, 151–161 10.3233/RNN-2009-0483 19531871

[B137] Suen Y. Chang M. Lee S. M. Buzby J. S. Cairo M. S. (1994). Regulation of interleukin-11 protein and mRNA expression in neonatal and adult fibroblasts and endothelial cells. Blood 84, 4125–4134 7527667

[B138] Sykova E. Jendelova P. Urdzikova L. Lesny P. Hejcl A. (2006). Bone marrow stem cells and polymer hydrogels-two strategies for spinal cord injury repair. Cell. Mol. Neurobiol. 26, 1113–1129 10.1007/s10571-006-9007-2 16633897 PMC11520705

[B139] Taguchi A. Soma T. Tanaka H. Kanda T. Nishimura H. Yoshikawa H. (2004). Administration of CD34+ cells after stroke enhances neurogenesis via angiogenesis in a mouse model. J. Clin. Invest. 114, 330–338 10.1172/JCI200420622 15286799 PMC484977

[B140] Takahashi S. Ooi J. Tomonari A. Konuma T. Tsukada N. Oiwa-Monna M. (2007). Comparative single-institute analysis of cord blood transplantation from unrelated donors with bone marrow or peripheral blood stem-cell transplants from related donors in adult patients with hematologic malignancies after myeloablative conditioning regimen. Blood 109, 1322–1330 17038536 10.1182/blood-2006-04-020172

[B141] Tanaka K. Nogawa S. Ito D. Suzuki S. Dembo T. Kosakai A. (2001). Activation of NG2-positive oligodendrocyte progenitor cells during post-ischemic reperfusion in the rat brain. Neuroreport 12, 2169–2174 10.1097/00001756-200107200-00025 11447328

[B142] Tohill M. Mantovani C. Wiberg M. Terenghi G. (2004). Rat bone marrow mesenchymal stem cells express glial markers and stimulate nerve regeneration. Neurosci. Lett. 362, 200–203 10.1016/j.neulet.2004.03.077 15158014

[B143] Ujike H. Takaki M. Kodama M. Kuroda S. (2002). Gene expression related to synaptogenesis, neuritogenesis, and MAP kinase in behavioral sensitization to psychostimulants. Ann. N. Y. Acad. Sci. 965, 55–67 10.1111/j.1749-6632.2002.tb04151.x 12105085

[B144] van Royen N. Piek J. J. Buschmann I. Hoefer I. Voskuil M. Schaper W. (2001). Stimulation of arteriogenesis; a new concept for the treatment of arterial occlusive disease. Cardiovasc. Res. 49, 543–553 10.1016/S0008-6363(00)00206-6 11166267

[B145] Vendrame M. Cassady J. Newcomb J. Butler T. Pennypacker K. R. Zigova T. (2004). Infusion of human umbilical cord blood cells in a rat model of stroke dose-dependently rescues behavioral deficits and reduces infarct volume. Stroke 35, 2390–2395 10.1161/01.STR.0000141681.06735.9b 15322304

[B146] Vendrame M. Gemma C. de Mesquita D. Collier L. Bickford P. C. Sanberg C. D. (2005). Anti-inflammatory effects of human cord blood cells in a rat model of stroke. Stem Cells Dev. 14, 595–604 10.1089/scd.2005.14.595 16305344

[B147] Vendrame M. Gemma C. Pennypacker K. R. Bickford P. C. Davis Sanberg C. Sanberg P. R. (2006). Cord blood rescues stroke-induced changes in splenocyte phenotype and function. Exp. Neurol. 199, 191–200 10.1016/j.expneurol.2006.03.017 16713598

[B148] Wakabayashi K. Nagai A. Sheikh A. M. Shiota Y. Narantuya D. Watanabe T. (2010). Transplantation of human mesenchymal stem cells promotes functional improvement and increased expression of neurotrophic factors in a rat focal cerebral ischemia model. J. Neurosci. Res. 88, 1017–1025 19885863 10.1002/jnr.22279

[B149] Walmsley A. R. Mir A. K. (2007). Targeting the Nogo-A signalling pathway to promote recovery following acute CNS injury. Curr. Pharm. Des. 13, 2470–2484 10.2174/138161207781368611 17692015

[B150] Wang L. Zhang Z. Wang Y. Zhang R. Chopp M. (2004). Treatment of stroke with erythropoietin enhances neurogenesis and angiogenesis and improves neurological function in rats. Stroke 35, 1732–1737 10.1161/01.STR.0000132196.49028.a4 15178821

[B151] Wang X. Q. Zhu X. J. Zou P. (2013). Research progress of mesenchymal stem cell-derived microvesicle. Zhongguo Shi Yan Xue Ye Xue Za Zhi 21, 227–230 10.7534/j.issn.1009-2137.2013.01.046 23484725

[B152] Watson D. J. Longhi L. Lee E. B. Fulp C. T. Fujimoto S. Royo N. C. (2003). Genetically modified NT2N human neuronal cells mediate long-term gene expression as CNS grafts in vivo and improve functional cognitive outcome following experimental traumatic brain injury. J. Neuropathol. Exp. Neurol. 62, 368–380 12722829 10.1093/jnen/62.4.368

[B153] Wei L. Erinjeri J. P. Rovainen C. M. Woolsey T. A. (2001). Collateral growth and angiogenesis around cortical stroke. Stroke 32, 2179–2184 10.1161/hs0901.094282 11546914

[B154] Weimar C. Kraywinkel K. Maschke M. Diener H. C. (2006). Intravenous thrombolysis in German stroke units before and after regulatory approval of recombinant tissue plasminogen activator. Cerebrovasc. Dis. 22, 429–431 10.1159/000094995 16912477

[B155] Wen Z. Zheng S. Zhou C. Yuan W. Wang J. Wang T. (2012). Bone marrow mesenchymal stem cells for post-myocardial infarction cardiac repair: microRNAs as novel regulators. J. Cell. Mol. Med. 16, 657–671 10.1111/j.1582-4934.2011.01471.x 22004043 PMC3822837

[B156] Weng J. S. Liu N. Du H. W. Chen R. H. Zhang Y. X. Wang J. H. (2008). Effects of bone marrow-derived mesenchymal stem cells transplantation on recovery of neurological functions and expression of synaptophysin in focal cerebral infarction in rats. Xi Bao Yu Fen Zi Mian Yi Xue Za Zhi 24, 34–37 18177615

[B157] Willing A. E. Lixian J. Milliken M. Poulos S. Zigova T. Song S. (2003). Intravenous versus intrastriatal cord blood administration in a rodent model of stroke. J. Neurosci. Res. 73, 296–307 10.1002/jnr.10659 12868063

[B158] Xin H. Li Y. Cui Y. Yang J. J. Zhang Z. G. Chopp M. (2013). Systemic administration of exosomes released from mesenchymal stromal cells promote functional recovery and neurovascular plasticity after stroke in rats. J. Cereb. Blood Flow Metab. 33, 1711–1715 10.1038/jcbfm.2013.152 23963371 PMC3824189

[B159] Xu J. Wu W. Zhang L. Dorset-Martin W. Morris M. W. Mitchell M. E. (2012). The role of microRNA-146a in the pathogenesis of the diabetic wound-healing impairment: correction with mesenchymal stem cell treatment. Diabetes 61, 2906–2912 10.2337/db12-0145 22851573 PMC3478555

[B160] Yan T. Chopp M. Ye X. Liu Z. Zacharek A. Cui Y. (2012). Niaspan increases axonal remodeling after stroke in type 1 diabetes rats. Neurobiol. Dis. 46, 157–164 10.1016/j.nbd.2012.01.001 22266016 PMC3335197

[B161] Yasuhara T. Hara K. Maki M. Xu L. Yu G. Ali M. M. (2010). Mannitol facilitates neurotrophic factor up-regulation and behavioural recovery in neonatal hypoxic-ischaemic rats with human umbilical cord blood grafts. J. Cell. Mol. Med. 14, 914–921 10.1111/j.1582-4934.2008.00671.x 20569276 PMC3823123

[B162] Yoo K. H. Jang I. K. Lee M. W. Kim H. E. Yang M. S. Eom Y. (2009). Comparison of immunomodulatory properties of mesenchymal stem cells derived from adult human tissues. Cell. Immunol. 259, 150–156 10.1016/j.cellimm.2009.06.010 19608159

[B163] Yuan M. Wen S. J. Yang C. X. Pang Y. G. Gao X. Q. Liu X. Q. (2013). Transplantation of neural stem cells overexpressing glial cell line-derived neurotrophic factor enhances Akt and Erk1/2 signaling and neurogenesis in rats after stroke. Chin. Med. J. 126, 1302–1309 23557563

[B164] Zacharek A. Chen J. Cui X. Li A. Li Y. Roberts C. (2007). Angiopoietin1/Tie2 and VEGF/Flk1 induced by MSC treatment amplifies angiogenesis and vascular stabilization after stroke. J. Cereb. Blood Flow Metab. 27, 1684–1691 10.1038/sj.jcbfm.9600475 17356562 PMC2796470

[B165] Zhang J. Li Y. Zhang Z. G. Lu M. Borneman J. Buller B. (2009). Bone marrow stromal cells increase oligodendrogenesis after stroke. J. Cereb. Blood Flow Metab. 29, 1166–1174 10.1038/jcbfm.2009.41 19384336 PMC2849641

[B166] Zhang J. H. Badaut J. Tang J. Obenaus A. Hartman R. Pearce W. J. (2012a). The vascular neural network – a new paradigm in stroke pathophysiology. Nat. Rev. Neurol. 8, 711–716 10.1038/nrneurol.2012.210 23070610 PMC3595043

[B167] Zhang L. Li Y. Zhang C. Chopp M. Gosiewska A. Hong K. (2011). Delayed administration of human umbilical tissue-derived cells improved neurological functional recovery in a rodent model of focal ischemia. Stroke 42, 1437–1444 10.1161/STROKEAHA.110.593129 21493915

[B168] Zhang L. Yi L. Chopp M. Kramer B. C. Romanko M. Gosiewska A. (2012b). Intravenous administration of human umbilical tissue-derived cells improves neurological function in aged rats after embolic stroke. Cell Transplant. 31, 31 10.3727/096368912X658674 23127976

[B169] Zhang R. Wang Y. Zhang L. Zhang Z. Tsang W. Lu M. (2002). Sildenafil (Viagra) induces neurogenesis and promotes functional recovery after stroke in rats. Stroke 33, 2675–2680 10.1161/01.STR.0000034399.95249.59 12411660

[B170] Zhang R. Zhang L. Zhang Z. Wang Y. Lu M. Lapointe M. (2001). A nitric oxide donor induces neurogenesis and reduces functional deficits after stroke in rats. Ann. Neurol. 50, 602–611 10.1002/ana.1249 11706966

[B171] Zhang R. L. Zhang Z. G. Lu M. Wang Y. Yang J. J. Chopp M. (2006). Reduction of the cell cycle length by decreasing G1 phase and cell cycle reentry expand neuronal progenitor cells in the subventricular zone of adult rat after stroke. J. Cereb. Blood Flow Metab. 26, 857–863 10.1038/sj.jcbfm.9600237 16251885

[B172] Zhang R. L. Zhang Z. G. Roberts C. LeTourneau Y. Lu M. Zhang L. (2008). Lengthening the G(1) phase of neural progenitor cells is concurrent with an increase of symmetric neuron generating division after stroke. J. Cereb. Blood Flow Metab. 28, 602–611 10.1038/sj.jcbfm.9600556 17928800 PMC2749512

[B173] Zhang Z. G. Chopp M. (2009). Neurorestorative therapies for stroke: underlying mechanisms and translation to the clinic. Lancet Neurol. 8, 491–500 10.1016/S1474-4422(09)70061-4 19375666 PMC2727708

[B174] Zhao L.-R. Duan W.-M. Reyes M. Keene C. D. Verfaillie C. M. Low W. C. (2002). Human bone marrow stem cells exhibit neural phenotypes and ameliorate neurological deficits after grafting into the ischemic brain of rats. Exp. Neurol. 174, 11–20 10.1006/exnr.2001.7853 11869029

[B175] Zhu C. J. Dong J. X. Li J. Zhang M. J. Wang L. P. Luo L. (2011). Preliminary study on the mechanism of acupoint injection of bone marrow mesenchymal stem cells in improving blood flow in the rat of hind limb ischemia. J. Tradit. Chin. Med. 31, 241–245 21977870 10.1016/s0254-6272(11)60050-2

